# Psychosis and substance abuse increase the COVID-19 mortality risk

**DOI:** 10.1017/S0033291722000976

**Published:** 2023-07

**Authors:** Ana Catalan, Claudia Aymerich, Amaia Bilbao, Borja Pedruzo, José Luis Pérez, Nerea Aranguren, Gonzalo Salazar de Pablo, Emily Hedges, Patxi Gil, Rafael Segarra, Ana González-Pinto, Aranzazu Fernández-Rivas, Lucía Inchausti, Philip McGuire, Paolo Fusar-Poli, Miguel Ángel González-Torres

**Affiliations:** 1 Osakidetza Basque Health Service, Department of Psychiatry, Basurto University Hospital, Bilbao, Spain; 2 Biocruces Bizkaia Health Research Institute, Barakaldo, Spain; 3 Facultad de Medicina y Odontología, University of the Basque Country, UPV/EHU, Leioa, Spain; 4 Department of Psychosis Studies, Institute of Psychiatry, Psychology & Neuroscience, King's College London, London, UK; 5 Osakidetza Basque Health Service, Basurto University Hospital, Research Unit, Bilbao, Bizkaia, Spain; 6 Health Service Research Network on Chronic Diseases (REDISSEC), Madrid, Spain; 7 Kronikgune Institute for Health Services Research, Barakaldo, Spain; 8 Unidad de Información OSI Bilbao-Basurto, Bilbao, Spain; 9 Early Psychosis: Interventions and Clinical-detection (EPIC) Lab, Department of Psychosis Studies, Institute of Psychiatry, Psychology & Neuroscience, King's College London, London, UK; 10 Department of Child and Adolescent Psychiatry, Institute of Psychiatry and Mental Health. Hospital General Universitario Gregorio Marañón, Madrid, Spain; 11 Bizkaia Mental Health Network, Programa Lehenak, Bilbao, Spain; 12 Psychiatry Department, Cruces University Hospital, Barakaldo, Spain; 13 Bioaraba. CIBERSAM. Psychiatry Department, Hospital Universitario de Alava, Vitoria-Gasteiz, Spain; 14 Outreach and Support in South London Service, South London and Maudsley National Health Service Foundation Trust, London, UK; 15 National Institute for Health Research Biomedical Research Centre, London, UK

**Keywords:** COVID-19, hospitalization, mental disorders, mortality, psychosis

## Abstract

**Background:**

The coronavirus disease 2019 (COVID-19) pandemic has been a global challenge. High mortality rates have been reported in some risk groups, including patients with pre-existing mental disorders.

**Methods:**

We used electronic health records to retrospectively identify people infected due to COVID-19 (between March 2020 and March 2021) in the three territories of the Basque Country. COVID-19 cases were defined as individuals who had tested positive on a reverse transcription-polymerase chain reaction (PCR) test. Univariate and multivariate logistic regression models and multilevel analyses with generalized estimated equations were used to determine factors associated with COVID-19-related mortality and hospital admission.

**Results:**

The COVID-19 mortality rate was increased for patients with psychotic disorders [odds ratio (OR) adjusted: 1.45, 95% confidence interval (CI) (1.09–1.94), *p* = 0.0114] and patients with substance abuse [OR adjusted: 1.88, 95% CI (1.13–3.14, *p* < 0.0152)]. The mortality rate was lower for patients with affective disorders [OR adjusted: 0.80, 95% CI (0.61–0.99), *p* = 0.0407]. Hospital admission rates due to COVID-19 were higher in psychosis [OR adjusted: 2.90, 95% CI (2.36–3.56), *p* < 0.0001] and anxiety disorder groups [OR adjusted: 1.54, 95% CI (1.37–1.72), *p* < 0.0001]. Among admitted patients, COVID-19 mortality rate was decreased for those with affective disorders rate [OR adjusted: 0.72, 95% CI (0.55–0.95), *p* = 0.0194].

**Conclusions:**

COVID-19-related mortality and hospitalizations rates were higher for patients with a pre-existing psychotic disorder.

## Introduction

The unprecedented coronavirus disease 2019 (COVID-19) outbreak has been a challenge for national health systems all over the world. Published research suggests that people with psychiatric disorders who tested positive for COVID-19 may be at greater risk of mortality and hospital admission (Liu et al., 2021; Nemani et al., 2021; Vai et al., 2021). Generally, it is well known that people with severe mental disorders have higher risks of premature death compared with the general population (Plana-Ripoll et al., 2019). This increased mortality may be explained by the comorbidity between psychiatric disorders and physical illness, e.g. diabetes (Foley et al., 2018), obesity (Chao, Wadden, & Berkowitz, 2019), cancer (Manderbacka et al., 2017), cardiovascular disease (Nielsen, Banner, & Jensen, 2021), and obstructive pulmonary disease (Partti et al., 2015). Besides, the lifestyle of patients with psychiatric disorders is often sedentary and deficient in self-care (Evert, Harvey, Trauer, & Herrman, 2003). During the outbreak, access to the different health services has been challenging for patients (Nunez, Sreeganga, & Ramaprasad, 2021), especially those with mental health problems (Aragona, Barbato, Cavani, Costanzo, & Mirisola, 2020). Telemedicine showed to be a useful tool in this situation (Abraham et al., 2021). People with mental disorders have difficulties accessing telemedicine (Costa et al., 2021), becoming an even more vulnerable population during the pandemic.

Several authors have reported an association between an increased risk of mortality due to COVID-19 infection and any psychiatric disorders (Barcella et al., 2021; Fond et al., 2021a; Maripuu, Bendix, Ohlund, Widerstrom, & Werneke, 2021; Nemani et al., 2021; Toubasi, AbuAnzeh, Tawileh, Aldebei, & Alryalat, 2021; Vai et al., 2021). Further, a recent meta-analysis found a higher COVID-19 mortality rate in people with schizophrenia spectrum disorders compared to the general population (Nemani et al., 2021). Other systematic reviews and meta-analyses have described similar odds ratios (ORs) for populations with psychotic disorders (Vai et al., 2021; Fond et al., 2021a). However, the relationship between COVID-19-related mortality and affective disorders is not yet clear. Compared to the general population, research has reported both similar (Nemani et al., 2021) and higher mortality rates (Vai et al., 2021) in people with mood or anxiety disorders. Diez-Quevedo et al. (Diez-Quevedo et al. 2021) described that delirium during hospital admission and a history of mood disorder was related to higher mortality risk, while psychotropic treatment in the previous year was associated with lower mortality risk. These results suggest that mental health problems may be a risk factor for mortality in patients with COVID-19 infection, although the nature of this relationship remains unclear. Moreover, it is not yet known whether the increased COVID-19 mortality is associated with psychotic disorders specifically, or with all mental health problems.

The present study aimed to (1) analyze the COVID-19 mortality rate over one year (from 14^th^ March 2020 to 14^th^ March 2021) for people with mental health disorders compared with the population without mental disorders; (2) evaluate the number of COVID-19-related hospitalizations in people with mental disorders; (3) report the COVID-19 mortality rate of patients with mental disorders who were admitted to hospital due to the infection, and (4) examine the influence of several sociodemographic and clinical variables on the COVID-19 mortality rate (sex, age, and physical comorbidity) in people with mental disorders. We hypothesized that the population with severe mental disorders would show worse outcomes (hospitalizations and mortality) than the general population without mental disorders.

## Material/subjects and methods

The Basque Country consists of three historical territories (Bizkaia, Gipuzkoa and Araba) in the North of Spain, with 2 199 711 million habitants (2020 EUSTAT- Euskal Estatistika Erakundea) (Estadística, 2021). We used an electronic health register (EHR; OBI- Unidad de Información OSI Bilbao-Basurto) to retrospectively identify people who tested positive for COVID-19 over one year (from 14^th^ of March 2020 to 14^th^ of March 2021) in the Basque Country. The Basque Health System, divided into 13 integrated healthcare organizations (IHOs), combines all primary and hospital care resources in given areas belonging to the three historical territories under the same administrative management (eTable 1). All subjects who suffered from COVID-19 infection were included. COVID-19 cases were defined as individuals who had tested positive on a reverse transcription-polymerase chain reaction (PCR) test. All the diagnostic tests were carried out in medical sites.

The rate of mortality and admissions due to COVID-19 were obtained through the EHR. We defined hospitalization due to COVID-19 as admissions for patients with positive tests whose COVID-19 diagnoses were dated concurrently or prior to admission (up to15 days), and mortality as deaths for patients with positive tests whose COVID-19 diagnoses were dated concurrently or prior to death (up to 2 months). Sociodemographic variables, physical illnesses and psychiatric pathologies were also documented. Physical and mental disorders were coded by DSM-IV (American Psychiatric Association, 2000), ICD-9 [World Health Organization(WHO), 1978] and ICD-10 [World Health Organization(WHO), 1993] classifications. People with severe mental disorders were divided into seven groups according to their primary diagnosis: psychotic disorders (schizophrenia, schizophrenia spectrum disorders, schizoaffective disorder, and psychotic no otherwise specified), affective disorders (bipolar disorder and depression), anxiety disorders, substance abuse, personality disorders, eating disorders, and other diagnoses (see eTable 2 for detailed descriptions).

The Basque Country Ethics Committee gave ethical approval for the study. The present study followed the REporting of studies Conducted using Observational Routinely-collected Data (RECORD) guideline for cohort studies (eTable 3).

### Statistical analyses

Descriptive statistics used included frequency tables for categorical variables and means, standard deviations (SDs), medians and interquartile ranges for continuous variables. First, univariate logistic regression models were employed to study the relationship between each sociodemographic characteristic (sex, age), physical comorbidities (cardiovascular disease, pulmonary, disease, metabolic and endocrine disease, renal disease, hepatic disease, neurology disease, obesity, and cancer) and mental disorder (substance abuse, anxiety disorders, psychosis, affective disorders, personality disorders, eating disorders, and others) with both mortality or hospital admission. In these models, mortality or hospital admission was included as the dependent variable and all patients' sociodemographic characteristics, physical comorbidities, and mental disorders as the independent variables. Then, multivariate analyses were performed by multilevel analyses with generalized estimated equations with a two-level structure: individual (patients) and the three historical territories of the Basque Country. Potential interactions between variables were also examined. In the final multivariate models, only factors with *p* < 0.05 were retained, except for the obesity. We decided to include this factor because it had been consistently related to a significantly increased risk of infection, hospitalization, severe disease mechanical ventilation, intensive care unit admission, and mortality relative to normal-weight patients (Cai, Yang & Zhang, 2021). ORs and 95% confidence intervals (CIs) were calculated. All statistical analyses were performed using SAS for Windows, version 9.4 (SAS Institute, Carey, NC) (Inc., 2013), and R© version 4.0.4 (Team, 2020).

## Results and discussion

### Sample

A total sample of 157 246 people from the 13 sites of the Basque Country (eTable 1) were COVID-19 positive during the study period; resulting in an infection rate of 7.1% of the total population. Of COVID-19 positive cases, 53.25% were female (*N* = 83 737). The most common age range was 40–49 years (16.63%; *N* = 26 153). The most common physical comorbidity in the sample was pulmonary disease (5.00%), followed by cancer (2.25%). Among all COVID-19 positive cases, 6.3% (*N* = 9898) presented with at least one psychiatric condition. The most prevalent disorders in this group were mood and anxiety disorders (2.83% and 2.44%, respectively). Sociodemographic and clinical characteristics of the sample are described in Table 1. A detailed description of the physical and mental disorders included in each category are listed in e Table 2, eTable 5 and eTable 6.

**Table 1.** Sociodemographic and clinical characteristics of the sample (*N* = 157 246)

Variables	*N* (%)	Mortality rate *N* (%)
Sex		
Male	73 509 (46.75)	1758 (2.39)
Female	83 737 (53.25)	1690 (2.02)
Age (years), mean (s.d.)	44.4 (23.3)	
Age (years) categorized		
⩽9	10 367 (6.59)	1 (0.01)
10–19	17 546 (11.16)	0
20–29	18 311 (11.64)	2 (0.01)
30–39	19 156 (12.18)	6 (0.03)
40–49	26 153 (16.63)	29 (0.11)
50–59	25 047 (15.93)	79 (0.32)
60–69	16 520 (10.51)	250 (1.51)
70–79	10 612 (6.75)	589 (5.55)
80–89	8971 (5.71)	1391 (15.51)
⩾90	4562 (2.90)	1101 (24.13)
Physical comorbidities		
Cardiovascular disease	3215 (2.04)	376 (8.58)
Pulmonary disease	7861 (5.00)	241 (3.07)
Metabolic and endocrine disease	2685 (1.71)	249 (9.27)
Renal disease	2366 (1.50)	486 (20.54)
Hepatic disease	487 (0.31)	37 (7.60)
Neurology disorder	1098 (0.70)	162 (14.75)
Obesity	1592 (1.01)	42 (2.64)
Cancer	3542 (2.25)	413 (11.66)
Psychiatric disorders		
Substance abuse	897 (0.57)	18 (2.01)
Anxiety disorder	3831 (2.44)	43 (1.12)
Psychosis	550 (0.35)	69 (12.55)
Affective disorder	4454 (2.83)	84 (1.89)
Personality disorders	23 (0.01)	0
Eating disorders	61 (0.04)	0
Others	708 (0.45)	37 (5.23)
Admissions to hospital	14 720 (9.36)	
Length of admission		
Mean (s.d.)	10.80 (13.56)	
Median (IQR)	7 (4–13)	

s.d., Standard deviation; IQR, Interquartile range.

Data is given as *N* (%) unless otherwise stated.

**Table 2.** Univariable and multivariable analysis for mortality risk among people who tested positive for COVID-19 (*N* = 157 246)

Variables	Univariable analysis	*p* value	Multivariable analysis with multilevel analysis	*p* value
	OR (95% CI)		OR (95% CI)	
Sex				
Male *v.* Female	1.19 (1.11–1.27)	0.0021	2.04 (1.88–2.20)	<0.0001
Age (years)	1.12 (1.11–1.12)	<0.0001	1.12 (1.12–1.13)	<0.0001
Physical comorbidities				
Cardiovascular disease	4.47 (3.93–5.08)	<0.0001		
Pulmonary disease	1.44 (1.26–1.65)	<0.0001		
Metabolic and endocrine disease	4.84 (4.23–5.54)	<0.0001	1.34 (1.14–1.56)	0.0003
Renal disease	13.26 (11.92–14.74)	<0.0001	1.99 (1.76–2.24)	<0.0001
Hepatic disease	3.70 (2.64–5.18)	<0.0001	1.54 (1.76–2.24)	0.0271
Neurology disorder	8.05 (6.79–9.55)	<0.0001		
Obesity	1.21 (0.89–1.65)	0.2228	1.72 (1.22–2,43)	0.0022
Cancer	6.55 (5.88–7.30)	<0.0001	2.33 (2.06–2.63)	<0.0001
Psychiatric disorders				
Substance abuse	0.91 (0.57–1.46)	0.4702	1.88 (1.13–3.14)	0.0152
Anxiety disorder	0.50 (0.37–0.68)	<0.0001		
Psychosis	6.51 (5.05–8.40)	<0.0001	1.45 (1.09–1.94)	0.0114
Affective disorder	0.85 (0.69–1.06)	0.1566	0.80 (0.61–0.99)	0.0407
Personality disorders	1.55 (0.21–11.49)	0.9435		
Eating disorders	<0.001 (<0.001, >999.9)	0.9082		
Others	2.47 (1.77–3.45)	<0.0001		

OR, Odds Ratio; CI, Confidence interval.

### COVID-19 mortality rate

Of the total sample, 2.19% of patients (*N* = 3448) died due to the COVID-19 infection. The COVID-19 mortality rate was higher for male patients compared to women (2.39% *v.* 2.02%, *p* < 0.001) and for older adults compared to children (⩽9 years 0.01% *v.* ⩾90 years 24.13%, *p* < 0.0001) (Table 1). Among physical comorbidities, higher COVID-19 mortality rates were associated with renal pathology (0.54%) and neurological illness (14.75%). Psychotic disorders had the highest COVID-19 mortality rates among psychiatric patients (12.55%). All COVID-19 mortality rates are detailed in Table 1.

Univariate analyses showed that male sex [OR 1.19, 95% CI (1.11–1.27), *p* = 0.0021], and older age [OR 1.12, 95% CI (1.11–1.12), *p* < 0.0001] were associated with increased COVID-19 mortality risk. Mortality risk was increased for all physical illnesses, except for obesity. Renal disease [OR 13.26, 95% CI (11.92–14.74), *p* < 0.0001], neurological disorders [OR 8.05, 95% CI (6.79–9.55), *p* < 0.0001], and cancer [OR 6.55, 95% CI (5.88–7.30), *p* < 0.0001] resulted in a higher risk of mortality (Table 2). Finally, multivariable analyses found that male sex [OR 2.04, 95% CI (1.88–2.20), *p* < 0.0001], older age [OR 1.12, 95% CI (1.12–1.13), *p* < 0.0001], metabolic and endocrine disease [OR 1.34, 95% CI (1.14–1.56), *p* = 0003), renal illness [OR 1.99, 95% CI (1.76–2.24), *p* < 0.0001], hepatic disease [OR 1.54, 95% CI (1.76–2.24)), obesity [OR: 1.72, 95% CI (1.22–2.43, *p* = 0.0022)), and cancer [2.33, 95% CI (2.06–2.63), *p* < 0.0001] were related to higher mortality risk. Supplementary analyses were performed using the body mass index (BMI) (BMI >30, obesity defined as having a BMI>30 kg/m^2^) (eTable 7).

Regarding psychiatric disorders, 2.38% of patients (*N* = 251) died due to COVID-19 infection. The mortality rate for patients with substance abuse diagnosis was 2.01%, anxiety disorders 1.12%, affective disorders 1.89%, personality disorder 0%, eating disorders 0% and others 5.23%. The highest mortality rate (12.55%) was associated with a psychotic disorder diagnosis. Univariable analyses found that patients with psychotic disorders had an OR of 6.51 [95% CI (5.05–8.40); *p* < 0.001] for COVID-19 mortality risk. This result remained significant in multivariable analyses [OR 1.45, 95% CI (1.09–1.94); *p* = 0.0114]. Patients with other psychiatric pathologies had increased COVID-19 mortality rates [OR 2.47, 95% CI (1.77–3.45); *p* < 0.001], while people with anxiety disorders had decreased rates [OR 0.50, 95% CI (0.37–0.68); *p* < 0.001]. However, in the multivariable analyses, these results did not remain statistically significant. COVID-19 mortality risk was increased for patients with substance abuse [OR 1.88, 95% CI (1.13–3.14); *p* = 0.0152], but decreased for patients with affective disorders [OR 0.80, 95% CI (0.61–0.99); *p* = 0.0138] (Table 2).

### Admissions to hospital due to COVID-19

From the total sample of COVID-19 positive cases, 14 720 patients were admitted to the hospital due to COVID-19 (9.36%) with a mean age of 44.4 (s.d.: 23.3) years and a mean length of stay of 10.80 (s.d.:13.56) days.

Univariable analyses showed that male sex [OR 1.45, 95% CI (1.40–1.50), *p* < 0.0001], older age [OR 1.06, 95% CI (1.05–1.06), *p* < 0.0001], and all physical illnesses were related to an increased possibility of hospital admission, particularly renal disease [OR 12.58, 95% CI (11.58–13.66), *p* < 0.0001], and metabolic and endocrine disease [OR 8.37, 95% CI (7.74–9.05), *p* < 0.0001] (Table 2). However, in the multivariable analyses, the findings for renal disease and obesity did not remain significant, while the highest hospital admission risk was for metabolic and endocrine disease [OR 3.47, 95% CI (3.18–3.79), *p* < 0.0001], and pulmonary disease [OR 2.97, 95% CI (2.77–3.17), *p* < 0.0001] (Table 3).

**Table 3.** Univariable and multivariable analysis for hospital admission among people who tested positive for COVID-19 (*N* = 157 246)

Variables	Univariable analysis	*p* value	Multivariable analysis with multilevel analysis	*p* value
	OR (95% CI)		OR (95% CI)	
Sex				
Male v. Female	1.45 (1.40–1.50)	<0.0001	1.79 (1.73–1.86)	<0.0001
Age (years)	1.06 (1.05–1.06)	<0.0001	1.05 (1.05–1.06)	<0.0001
Physical comorbidities				
Cardiovascular disease	6.73, (6.26–7.24)	<0.0001	1.85 (1.70–2.01)	<0.0001
Pulmonary disease	2.88 (2.72–3.05)	<0.0001	2.97 (2.77–3.17)	<0.0001
Metabolic and endocrine disease	8.37 (7.74–9.05)	<0.0001	3.47 (3.18–3.79)	<0.0001
Renal disease	12.58 (11.58–13.66)	<0.0001		
Hepatic disease	6.48 (5.40–7.78)	<0.0001	2.93 (2.37–3.63)	<0.0001
Neurology disorder	7.17 (6.36–8.10)	<0.0001	1.53 (1.34–1.74)	<0.0001
Obesity	3.21 (2.86–3.61)	<0.0001		
Cancer	5.71 (5.32–6.14)	<0.0001	2.28 (2.10–2.47)	<0.0001
Psychiatric disorders				
Substance abuse	1.26 (1.02–1.55)	0.0289		
Anxiety disorder	1.15 (1.04–1.28)	0.0066	1.54 (1.37–1.72)	<0.0001
Psychosis	7.00 (5.90–8.30)	<0.0001	2.90 (2.36–3.56)	<0.0001
Affective disorder	1.69 (1.55–1.84)	<0.0001		
Personality disorders	3.42 (1.35–8.67)	0.0096		
Eating disorders	0.33 (0.08–1.34)	<0.0001		
Others	2.02 (1.66–2.46)	<0.0001		

OR, Odds Ratio; CI, Confidence interval.

All psychiatric pathologies were associated with an increased hospitalization rate in univariable analyses. In the multivariable analyses, only patients with psychotic disorders [OR 2.90, 95% CI (2.36–3.56), *p* < 0.0001], and patients with anxiety disorders [OR 1.54, 95% CI (1.37–1.72), *p* < 0.0001] had higher risks of hospital admission (Table 3).

### COVID-19 mortality rate in patients admitted to hospital

For patients who were admitted to the hospital due to COVID-19 infection, older adults [OR 1.09, 95% CI (1.08–1.09), *p* < 0.0001] had increased COVID-19 mortality risk in the univariable analyses. All physical illnesses were related to higher mortality rates, except for cardiovascular disease, metabolic and endocrine disease, and hepatic disease. Among psychiatric comorbidity, patients with psychotic disorders [OR 1.65, 95% CI (1.21–2.26), *p* = 0015] and patients with other psychiatric disorders [OR 1.63, 95% CI (1.07–2.49), *p* < 0.0099] had higher mortality risk (Table 4).

**Table 4.** Univariable and multivariable analysis for mortality risk among patients with COVID-19 who were admitted to hospital (*N* = 14 720)

Variables	Univariable analysis	*p* value	Multivariable analysis with multilevel analysis	*p* value
	OR (95% CI)		OR (95% CI)	
Sex				
Male v. Female	1.80 (0.99–1.18)	0.0919	1.55 (1.40–1.72)	<0.0001
Age (years), mean (s.d.)	1.09 (1.08–1.09)	<0.0001	1.09 (1.09–1.10)	<0.0001
Physical comorbidities				
Cardiovascular disease	1.15 (0.98–1.24)	0.0817	0.58 (0.50–0.68)	<0.0001
Pulmonary disease	0.67 (0.57–0.78)	<0.0001	0.68 (0.57–0.81)	<0.0001
Metabolic and endocrine disease	1.10 (0.93–1.28)	0.2607		
Renal disease	2.68 (2.36–3.04)	<0.0001	1.50 (1.30–1.72)	<0.0001
Hepatic disease	1.12 (0.77–1.63)	0.5649		
Neurology disorder	2.04 (1.65–2.52)	<0.0001		
Obesity	0.53 (0.37–0.75)	0.0003		
Cancer	2.10 (1.84–2.40)	<0.0001	2.04 (1.76–2.36)	<0.0001
Psychiatric disorders				
Substance abuse	0.85 (0.49–1.50)	0.5875		
Anxiety disorder	0.59 (0.42–0.81)	0.0015		
Psychosis	1.65 (1.21–2.26)	0.0015		
Affective disorder	0.68 (0.53–0.87)	0.0020	0.72 (0.55–0.95)	0.0194
Personality disorders	<0.001 (<0.001, >999.9)	0.9494		
Eating disorders	<0.001 (<0.001, >999.9)	0.9565		
Others	1.63 (1.07–2.49)	0.0243		

OR, Odds Ratio; CI, Confidence interval.

Multivariable analyses found that male sex [OR 1.55, 95% CI (1.40–1.72), *p* < 0.0001], older age [OR 1.09, 95% CI (1.09–1.10), *p* < 0.0001], renal disease [OR 1.50, 95% CI (1.30–1.72), *p* < 0.0001], and cancer [OR 2.04, 95% CI (1.76–2.36), *p* < 0.0001] were associated with higher mortality risk. In psychiatric comorbidity, the mortality risk was lower for patients with affective disorders [OR: 0.72, 95% CI (0.55–0.95), *p* = 0.0124] (Table 4).

## Discussion

Our study found an increased COVID-19 mortality risk for patients with a pre-existing psychotic and substance abuse disorder. Affective disorders were related to decreased mortality risk compared to those without an affective disorder. We found no relationship between mortality risk and other psychiatric disorders. As expected, other sociodemographic and clinical variables were also associated with increased COVID-19 mortality risk, such as male sex, age and physical comorbidity (metabolic and endocrine disease, renal illness, hepatic disease, obesity, and cancer). The risk of hospital admissions due to COVID-19 was higher in the anxiety disorders and psychotic disorders groups. Among those admitted to the hospital, patients with affective disorders presented a decreased COVID-19 mortality risk

Although the association of physical comorbidity and increased COVID-19 mortality risk has been widely reported (Espana et al., 2021), the relationship with psychiatric disorders is less clear. A recent meta-analysis (Vai et al., 2021) showed that the existence of any pre-existing mental illness was related to an increased risk of COVID-19 mortality [OR 2.00, 95% CI (1.58–2.54)], although other authors (Fond et al., 2021a) found lower ORs [OR 1.38, 95% CI (1.15–1.65)]. However, in both reviews, patients with psychotic disorders presented the highest mortality rates, while anxiety disorders were not associated with higher mortality rates (Fond et al., 2021a; Vai et al., 2021). These findings are in line with the results of the present study, where the increased COVID-19 mortality risk for patients with psychotic disorders was 1.45, 95% CI (1.09–1.94). Although prior evidence of a relationship between COVID-19 mortality risk and affective disorders is mixed (Nemani et al., 2021; Vai et al., 2021), we found a decreased mortality risk in this group, including bipolar disorder (OR: 0.76 (0.62, 0.95)).

In our study, hospitalizations were higher for anxiety disorder and psychotic patients; the latter of whom had a 3-fold increased probability of hospitalization compared to patients without psychotic disorders. In a recent study, individuals with schizophrenia were less likely to test positive for COVID-19; however, they were twice as likely to be hospitalized for COVID-19 [OR 2.15, 95% CI (1.63–2.82); *p* < 0.0001], even after controlling for sociodemographic and clinical risk factors [OR 1.88, 95% CI (1.39–2.55); *p* < 0.0001] (Bitan et al., 2021).

Increased COVID-19 mortality risk has consistently been found in patients with psychiatric disorders compared to people without psychiatric pathology (Bitan et al., 2021; Fond et al., 2021a, 2021b; Lee et al., 2020; Li, Li, Fortunati, & Krystal, 2020; Maripuu et al., 2021; Wang, Xu, & Volkow, 2021). In our study, this increase in mortality was mainly associated with psychosis and substance use disorders. Similarly, previous studies have described increased mortality in patients with substance use disorders (Wang, Kaelber, Xu, & Volkow, 2021). As it is well known that patients with psychotic disorders have higher all-cause mortality (Walker, McGee, & Druss, 2015), it is not surprising that psychosis is a likely contributing factor to mortality following COVID-19 infection. An increased risk of COVID-19 mortality in patients with psychotic disorders could reflect biological processes, such as immunoinflammatory alterations (Barron, Hafizi, Andreazza, & Mizrahi, 2017) including immunogenetic abnormalities, elevated levels of cytokines, autoantibodies, and aberrant counts of leukocyte cell types that characterize psychiatric disorders (Radhakrishnan, Kaser, & Guloksuz, 2017). Other factors, such as an impairment in social functioning and lifestyle (e.g. diet, physical inactivity, social isolation, and high substance use) and a higher prevalence of somatic comorbidities (e.g. diabetes, cardiovascular disease, and respiratory disease) (Stubbs et al., 2016) may also have adverse effects on the prognosis of COVID-19 infection in this group of patients. Recent studies (Hoertel et al., 2022; Sanchez-Rico, Limosin, & Hoertel, 2022) have associated the obesity excess described in psychosis with higher mortality rates. Nevertheless, we have found that COVID-19 mortality in psychosis is not affected by this factor in our study. Reduced access to care has been reported for nearly every aspect of somatic health care in patients with psychotic disorders (Kular et al., 2019; Lawrence & Kisely, 2010). Further to this, socioeconomic disadvantages have also been associated with an increased risk of COVID-19 mortality (Karmakar, Lantz, & Tipirneni, 2021; Seligman, Ferranna, & Bloom, 2021), and the relationship between psychosis and socioeconomic disadvantage has been recognized across diverse cultural, social, and demographic contexts (Sweeney, Air, Zannettino, & Galletly, 2015).

Depression has been reported as a risk factor for hospitalization due to COVID-19 (OR 6.06; 95% CI .54–40.42) (Siso-Almirall et al., 2020). However, its relationship to COVID-19 mortality risk is less clear. While some studies have described an increased mortality risk (Ceban et al., 2021; Vai et al., 2021), others did not find this association (Nemani et al., 2021). The present study found a decreased mortality risk in this group, suggesting that patients with depression may be more likely seek help sooner than patients with psychotic disorders. In fact, depression is associated with a more urgent use of health care resources (Dickens, Cherrington, & McGowan, 2012). Further, Hoerthel et al. (Hoertel et al. 2021) reported a significant association between antidepressant use and reduced risk of intubation or death [HR 0.56, 95% CI (0.43–0.73), *p* < 0.001], which may indicate that antidepressant treatment could be associated with lower risk of death in patients hospitalized for COVID-19.

This study consisted of a large sample of patients recruited using the same healthcare system over a one-year period. Previous studies are limited to shorter follow-up (Nemani et al., 2021). However, this study has several limitations. First, psychotropic medication use was not considered in our analyses in the present study. Although some studies have found that vulnerable severe mental disorder individuals on antipsychotic treatment had a lower risk of severe acute respiratory syndrome coronavirus 2 (SARS-CoV2) infection and a better COVID-19 prognosis (Canal-Rivero et al., 2021), these findings should be interpreted cautiously as there are difficulties determining medication adherence in such large samples. A second limitation is that not all diagnoses were well-recorded in the EHR (Yolken, 2021) or the lack of information about the stability or phase of the reported illnesses. Third, data used in the present study did not include COVID-19 treatment information, which is likely to have influenced mortality risk. A fourth limitation is that the COVID-19 prevalence may have been underestimated as some infected people may have not had a PCR test. In addition, the mortality rate can be overestimated due to the same reason.

The COVID-19 pandemic has affected the mental health of the general population, vulnerable psychiatric groups and healthcare professionals (Salazar de Pablo et al., 2020). Moreover, the physical and mental health of patients affected with mental disorders is further compromised (Fusar-Poli, Brambilla, & Solmi, 2020). Furthermore, there may be longer-term adverse outcomes for all people following COVID-19 infection, such as risks of depression, anxiety, fatigue, post-traumatic stress disorder, and rarer neuropsychiatric syndromes (Rogers et al., 2020).

Research that focuses on the mental health of populations is often considered a second-order priority. We strongly believe that increased knowledge of the consequences of the COVID-19 outbreak on population with mental disorders is important to develop strategies to protect vulnerable groups. For example, health strategy recommendations after the COVID-19 pandemic for patients with early-onset psychosis have been recently published (Jauhar et al., 2021). Furthermore, most European countries, including Spain, have not included psychiatric disorders as risk comorbidities eligible for vaccine prioritization (España, 2021), which could lead to detrimental outcomes for these individuals and their communities (De Picker, Dias, et al., 2021a). In our opinion, the psychiatric population, and especially patients with psychotic disorders and substance abuse, should be prioritized in the vaccination programs (De Hert, Mazereel, Detraux, & Van Assche, 2021; De Picker, Yolken, et al., 2021b; Mazereel, Van Assche, Detraux, & De Hert, 2021).

## Supplementary material

For supplementary material accompanying this paper visit https://doi.org/10.1017/S0033291722000976. click here to view supplementary material
