# Supplementary material for: Psychosis and substance abuse increase the COVID-19 mortality risk
Source: Psychol Med. 2022 Apr 12;53(9):4236–44. doi: 10.1017/S0033291722000976 (PMC9114752; doi:10.1017/S0033291722000976)
Supplement: Supplementary file 1 [file S0033291722000976sup001.docx]

Supplementary material

[eTable 1. Sites included in the sample 2](#_Toc98927432)

[eTable 2. Pathology codes ICD-9, ICD-10, and DSM-IV 2](#_Toc98927433)

[eTable 3. The RECORD statement – checklist of items, extended from the STROBE statement, that should be reported in observational studies using routinely collected health data. 5](#_Toc98927434)

[eMETHODS. Supplementary analyses with Body Index Mass (BIM). 11](#_Toc98927435)

[eTable 5. Descriptive data with BMI. 11](#_Toc98927436)

[eTable 6. Description of mortality and BMI; p<0.0001. 11](#_Toc98927437)

[eTable 7. Multilevel analysis including BMI. 11](#_Toc98927438)

# eTable 1. Sites included in the sample

| Basque Country historical terrioties and integrated healthcare organizations (IHOs) | N | % |
| --- | --- | --- |
| Bizkaia Historical Territory |  |  |
| IHO Barakaldo Sestao | 8,963 | 5,70 |
| IHO Barrualde-Galdakao | 18,810 | 11,96 |
| IHO Bilbao-Basurto | 27,611 | 17,56 |
| IHO Ezkerraldea Enkarterri Cruces | 10,498 | 6,68 |
| IHO Uribe | 14,077 | 8,95 |
| Gipuzkoa Historical Territory |  |  |
| IHO Alto Deba | 5,906 | 3,76 |
| IHO Bidasoa | 4,649 | 2,96 |
| IHO Debabarrena | 6,739 | 4,29 |
| IHO Donostialdea | 24,332 | 15,47 |
| IHO Goierri-Alto Urola | 7,760 | 4,93 |
| IHO Tolosaldea | 4,883 | 3,11 |
| Araba Historical Territory |  |  |
| IHO Araba | 22,283 | 14,17 |
| IHO Arabako Errioxa | 735 | 0,47 |

# eTable 2. Pathology codes ICD-9, ICD-10, and DSM-IV

| Disease |  | Code |
| --- | --- | --- |
| Cardiovascular disease | Valvular pathology | 396.8; 424.1; 424.0; 394-396; 395.2; 396.3; 395.1; 394.1 |
|  | Aneurysms | 442.81; 441.1; 441.2; 441.4; 441.9; 441; 437.3; 442.0; 442.3; 442.83; 442.9 |
|  | Arrhythmias | 426.9; 427.31; 427.3; 426.2; 427.89; 426.13; 426.53; 426.3; 427.2; 427.0; 427.32; 426.XX; 427.9 |
|  | Angina | 413; I200; I201; I209; I208 |
|  | Coronary atherosclerosis | 414.01; 414.00 |
|  | Intermittent claudication | 440.21 |
|  | Heart disease aortic dissection | 441.0; 441.00 |
|  | Endocarditis | 424.9 |
|  | Cardiac disease | 429.9; 411.1; 404.91; 404.90; 402.10; 402.91; 402.91; 402.91; 402.91; 402.90; 402.90; 402; I110; I119; 414.9; I130; 416.9; 436 |
|  | Pericardium | 423.9 |
|  | HTA | 401 |
|  | IAM | 410.71; 410.10; I21; I2111; I2119; I2109; I213; I2129; I214; 410; 412; I21A9 |
|  | Heart failure | 428; 428.41; 428.0; 428.1; 428.30; 428.20; 428.43 |
|  | Cardiomyopathy | 425; 425.11; 425.18; 425.4 |
|  | Ischemic disease | 437.1; 411; 411.8; 414.8 |
| Pulmonary disease | Asbestosis | 501 |
|  | Asthma | 493.XX |
|  | Pneumonia | 485; 482.9; 484.8; 484; 481; 486; 482.3; 480.8; 480.1; 482 |
|  | Bronchiectasis | 494.XX |
|  | Bronchitis | J42; 490; 491.XX; J410 (includes COPD) |
|  | Lung collapse | 518.0 |
|  | Pleural effusion | 511.9; 511.89; 518.4 |
|  | Pulmonary embolism | 416.2; 415.1 |
|  | Emphysema | J432; 518.1; J439; 492.8 |
|  | Fibrosis | 516.31; 515 |
|  | Respiratory failure | 518.81; 518.84; 518.83 |
|  | Flu | 487; 487.0; 487.8; 487.1; 488.1; 488.82 |
|  | Pulmonary HT | 416.0 |
|  | Corona pneumonia | 480.3 |
|  | Pulmonary infarction | 415.19 |
|  | Others | 518; 518.89; 518.82; 49 |
| Endocrine metabolic disorders | Diabetes | 250; E1010; E108; E109; E10620; E1169; E118: E119; E1065; E10649; E1021; E1042; E1144; E1151; E1110; E1165; E1100; E11641; E1121; E1140; E1169; E1129; E1139; E1142; E11319; E11359; E11621; E119; 249.30; 249.00 |
| Renal pathology | Renal vascular disorder | 593.81 |
|  | Kidney disease | 403.90; I129; I120; N181; N182; N183; N184; N185; N189; N186; N179; 584; 584.9; 586; N19 |
|  | Nephritis | 582.9; 582.2 |
|  | Nephropathy | 583.2; 583.9; 585.2; 585.4; 585.9 |
| Hepatic disease | Cirrhosis | K7030; K743; K7460; K761; K7469 |
|  | Hepatic disease | K709; K769; K759; K740; K7200; K7291; K7290 |
|  | Hepatitis | K754; K739 |
|  | Other | K7689 |
| Neurological disease | Dementia | 290.0; 290.1; 290.12; 290.13; 290.20; 290.21; 290.3; 290.40; 290.41; 294.1; 294.11; 294.10; 294.21; 438.0; |
|  | Delirium | 291.0; 293.1; 293.0 |
|  | Cerebral infarction | 434.11; 435; 435.9; 434.91; 434.01 |
|  | Hemiplegia | 438.20 |
|  | Haemorrhage | 431; 432.9; 430; 432.1 |
|  | Others | 437.9; 416.8; 435.8 |
| Obesity |  | E662; E6601; E669; E663 |
| Cancer | Lung cancer | 231.2 |
|  | Liver carcinoma | C220; C221; C227 |
|  | Esophageal carcinoma | 230.1 |
|  | Breast carcinoma | 230.0 |
|  | Bladder carcinoma | 233.7 |
|  | Cervical carcinoma | 233.1 |
|  | Colon carcinoma | 230.3 |
|  | Hodgkin | 201.90; 201.40 |
|  | Leukemia | C9101; C9100; C9110; 204.00; 204.10; 204.90; C9190; 205.10; C9210; 208.90; C9590; 205.80; C92Z0 |
|  | Lymphoma | C8370; C8310; C8319; C8190; 200.30; C8338; C8330; C8339; C8233; C8290; C8299; 202.80; 202.00; C9151; C9150; C8380; C8389; C8582; C8580; C8589 |
|  | Melanoma | 172.9; C4372; C4370; C4359; C4330; 172.7; C439 |
|  | Mesothelioma | C450; C459 |
|  | Myeloma | 203.00; C9001; C9000 |
|  | Others | 235.0; 236.2; 236.4; 236.7; 153.3; 156.2; 151.2; 162.9; 191.9; 180.9; 153.4; 153.6; 153.9; 153.1; 170.2; 155.1; 174.4; 182.0; 180.0; 151.9; 151.3; 194.0; 197.7; 155.2; 155.0; 161.9; 174.9; 170.1; 189.9; 149.8; 174.8; 183.0; 157.9; 195.3; 163.9; 154.1; 158.0; 189.0; 188.9; 156.0; 162.3; 186.9; 198.3; 198.5; 197.3; 196.9; 171.9; 160.0; 197.0; C801; C762; C099; C241; C185; C183; C163; C01; C069; C3400; C34; C250; C760; C716; C719; C180; C182; C186; C187; C184; C189; C412; C50419; C539; C530; C541; C159; C169; C140; C161; C07; 193; C73; C320; C229; C228; 148.9; C139; C419; C414; C179; 145.9; C029; 146.9; 145.4; 140.9; C002; C329; 141.9; C3432; C3430; C3411; C3410; C712; C50912; C50919; C718; C148; C50819; C411; C962; C383; C542; 147.9; C119; C689; C109; C257; C569; C259; C7490; C3491; C3490; C763; C659; C609; C164; C61; C20; C480; C641; C642; C649; 144.9; C049; C321; C490; C495; C499; C6210; C6290; C19; C669; C55; C679; C23; C240; C519; C800; 185; C7931; C7970; C787; C7951; C781; C7952; C7889; C7960; C792; C782; C7801; C7800; C786; C773; C770; C771; C779; 173.9; 199.1; 209.21; C7A012; C7A095; 209; C7A1 |
| Substance abuse |  | 305.XX; F12122; F1210; F1410; F1810; F11122; F1110; F1510; F1310; F1590; 304.32; F14222; F1420; 304; F1120; F152; 303.90; 305.90; 292.9; 305.1; F17200; F172 |
| Anxiety disorders | Anxiety | 300; 309.29; 309.89; 306.8; 300.09; F41; F413; F439; 308.9; F430; 313.0; F411; 300.02; 309.21; F419 |
|  | Panic disorder | F410; 300.01 |
|  | Agoraphobia | 300.21; F4002; F4000; 300.22 |
|  | Claustrophobia | F40240 |
|  | Organic anxiety disorder | 306.XX |
|  | Phobias | 300.20; 300.23; F4011; 300.29; F40248 |
|  | Hypochondria | 300.7; F4521 |
|  | Insomnia | 307.47; 307.49; 307.46; 307.40; 307.41; 307.4 |
|  | Reactions to stress | F438; 308.3 |
|  | Somatoform disorders | F458; F459 |
|  | Dissociative Disorder | F449 |
|  | TOC | F42; 300.3; 307.53 |
|  | Post-traumatic stress disorder | 309.81; F4311; F4310 |
| Psychosis | Autism | 299.00 |
|  | Schizophrenia | F20.0; 295.32; F20.5; F20.9 |
|  | Psychosis | 299.80; 298.8; 298.9; F29; 299.90 |
|  | Delusional disorder | 297.1; F22 |
|  | Schizoaffective disorder | F259 |
|  | Brief psychotic disorder | F23 |
| Affective disorders | Depression | F338; 296.99; 309.1; 309; 309.9; 309.24; 296.34; F4324; F4322; F4321; F4323; F4320; 309.0; 309.28; 313.1; 296.82; 296.30; F323; F329; F33; F333; F339; 311 |
|  | Dysthymia | 300.4; F341 |
|  | Bipolar disorder | F3010; 296.89; F308; F4320; 296.62; F3181; F3130; F3110; F3160; F319; 296.80; F319; 296.81; 296.00 |
| Personality disorders |  | 301.89; 301.9; 301.2; 301.83 |
| Eating disorders |  | 307.51; 307.59; 307.5; 307.50 |
| Others | Gambling | 312.31 |
|  | Another mixed emotional disturbance of adolescence | 313.89; 313 |
|  | Impulse control disorder | 312.39; 312.30 |
|  | Persistent mental disorders | 294.8 |
|  | Other conduct disorders | 312.89 |
|  | Behavioural disturbance, unspecified | 312.9 |
|  | ADHD | 314.01; 314.00 |
|  | Socialized Behaviour disorder | 312.21; 312.20; 312.82; 312.81 |
|  | Emotional disorder of childhood or adolescence NOS | 313.9 |
|  | Non-psychotic mental disorder, unspecified | 300.9; F489 |
|  | Persistent mental disorders, unspecified | 294.9 |

# eTable 3. The RECORD statement – checklist of items, extended from the STROBE statement, that should be reported in observational studies using routinely collected health data.

|  | **Item No.** | **STROBE items** | **Location in manuscript where items are reported** | **RECORD items** | **Location in manuscript**  **where items are reported** |
| --- | --- | --- | --- | --- | --- |
| **Title and abstract** | | | | | |
|  | 1 | (a) Indicate the study’s design with a commonly used term in the title or the abstract (b) Provide in the abstract an informative and balanced  summary of what was done and what was found | Title | RECORD 1.1: The type of data used should be specified in the title or  abstract. When possible, the name of the databases used should be included.  RECORD 1.2: If applicable, the geographic region and timeframe within which the study took place should be reported in the title or  abstract.  RECORD 1.3: If linkage between databases was conducted for the study, this should be clearly stated in the title or abstract. | Title |
| **Introduction** | | | | | |
| Background rationale | 2 | Explain the scientific background and rationale for the investigation being reported | Introduction |  |  |
| Objectives | 3 | State specific objectives, including any prespecified  hypotheses | Introduction |  |  |
| **Methods** | | | | | |
| Study Design | 4 | Present key elements of study design early in the paper | Methods |  |  |
| Setting | 5 | Describe the setting, locations, and relevant dates, including  periods of recruitment, exposure, follow-up, and data collection | Methods |  |  |
| Participants | 6 | *(a) Cohort study* - Give the |  | RECORD 6.1: The methods of study |  |
|  |  | eligibility criteria, and the  sources and methods of selection of participants. Describe methods of follow-up  *Case-control study* - Give the eligibility criteria, and the  sources and methods of case ascertainment and control  selection. Give the rationale for the choice of cases and controls *Cross-sectional study* - Give the eligibility criteria, and the  sources and methods of selection of participants  *(b) Cohort study* - For matched studies, give matching criteria and number of exposed and unexposed  *Case-control study* - For matched studies, give matching criteria and the number of  controls per case | Methods | population selection (such as codes or algorithms used to identify subjects) should be listed in detail. If this is not possible, an explanation should be provided.  RECORD 6.2: Any validation studies of the codes or algorithms used to  select the population should be referenced. If validation was conducted for this study and not published  elsewhere, detailed methods and results should be provided.  RECORD 6.3: If the study involved linkage of databases, consider use of a flow diagram or other graphical display to demonstrate the data linkage process, including the number of individuals with linked data at each  stage. | Methods |
| Variables | 7 | Clearly define all outcomes,  exposures, predictors, potential confounders, and effect modifiers. Give diagnostic  criteria, if applicable. |  | RECORD 7.1: A complete list of codes and algorithms used to classify  exposures, outcomes, confounders, and effect modifiers should be provided. If these cannot be reported, an  explanation should be provided. | Methods |
| Data sources/ measurement | 8 | For each variable of interest, give sources of data and details of methods of assessment (measurement).  Describe comparability of  assessment methods if there is more than one group | Methods |  | Methods |
| Bias | 9 | Describe any efforts to address  potential sources of bias | Methods |  | Methods |
| Study size | 10 | Explain how the study size was  arrived at | Methods |  | Methods |
| Quantitative variables | 11 | Explain how quantitative variables were handled in the  analyses. If applicable, describe which groupings were chosen, and why | Methods |  | Methods |
| Statistical methods | 12 | 1. Describe all statistical methods, including those used to control for confounding 2. Describe any methods used to examine subgroups and interactions 3. Explain how missing data were addressed 4. *Cohort study* - If applicable, explain how loss to follow-up was addressed   *Case-control study* - If applicable, explain how  matching of cases and controls was addressed  *Cross-sectional study* - If  applicable, describe analytical methods taking account of  sampling strategy   1. Describe any sensitivity analyses | Methods |  |  |
| Data access and  cleaning methods |  | .. |  | RECORD 12.1: Authors should  describe the extent to which the investigators had access to the database population used to create the study population.  RECORD 12.2: Authors should provide information on the data  cleaning methods used in the study. | Methods |
| Linkage |  | .. |  | RECORD 12.3: State whether the |  |
|  |  |  |  | study included person-level, institutional-level, or other data linkage across two or more databases. The methods of linkage and methods of linkage quality evaluation should be  provided. | Methods |
| **Results** |  |  |  |  |  |
| Participants | 13 | 1. Report the numbers of individuals at each stage of the study (*e.g.*, numbers potentially   eligible, examined for eligibility, confirmed eligible, included in the study, completing follow-up, and analysed)   1. Give reasons for non- participation at each stage. 2. Consider use of a flow diagram | Results | RECORD 13.1: Describe in detail the selection of the persons included in the study (*i.e.,* study population selection) including filtering based on data quality, data availability and linkage. The selection of included persons can be described in the text and/or by means of the study flow diagram. | Results |
| Descriptive data | 14 | 1. Give characteristics of study participants (*e.g.*, demographic, clinical, social) and information on exposures and potential   confounders   1. Indicate the number of participants with missing data for each variable of interest 2. *Cohort study* - summarise follow-up time (*e.g.*, average and total amount) | Results |  | Results |
| Outcome data | 15 | *Cohort study* - Report numbers  of outcome events or summary measures over time  *Case-control study* - Report numbers in each exposure  category, or summary measures of exposure  *Cross-sectional study* - Report numbers of outcome events or | Results |  |  |
|  |  | summary measures | Results |  |  |
| Main results | 16 | 1. Give unadjusted estimates and, if applicable, confounder- adjusted estimates and their precision   (e.g., 95% confidence interval). Make clear which  confounders were adjusted for and why they were included   1. Report category boundaries when continuous variables were categorized 2. If relevant, consider translating estimates of relative risk into absolute risk for a meaningful time period | Results |  |  |
| Other analyses | 17 | Report other analyses done—e.g., analyses of  subgroups and interactions, and sensitivity analyses | Results |  |  |
| **Discussion** | | | | | |
| Key results | 18 | Summarise key results with  reference to study objectives | Discussion |  |  |
| Limitations | 19 | Discuss limitations of the study, taking into account sources of potential bias or imprecision.  Discuss both direction and magnitude of any potential bias | Discussion | RECORD 19.1: Discuss the implications of using data that were not created or collected to answer the  specific research question(s). Include discussion of misclassification bias, unmeasured confounding, missing data, and changing eligibility over time, as they pertain to the study being reported. | Discussion |
| Interpretation | 20 | Give a cautious overall interpretation of results considering objectives,  limitations, multiplicity of  analyses, results from similar studies, and other relevant  evidence | Discussion |  |  |
| Generalisability | 21 | Discuss the generalisability (external validity) of the study  results | Discussion |  |  |
| **Other Information** |  |  |  |  |  |
| Funding | 22 | Give the source of funding and the role of the funders for the present study and, if applicable, for the original study on which  the present article is based | Funding |  |  |
| Accessibility of protocol, raw data, and programming  code |  | .. |  | RECORD 22.1: Authors should provide information on how to access any supplemental information such as the study protocol, raw data, or  programming code. | Supplementary material |

*Reference: Benchimol EI, Smeeth L, Guttmann A, Harron K, Moher D, Petersen I, Sørensen HT, von Elm E, Langan SM, the RECORD Working Committee. The REporting of studies Conducted using Observational Routinely-collected health Data (RECORD) Statement. *PLoS Medicine* 2015; in press

# eMETHODS. Supplementary analyses with Body Index Mass (BIM).

## eTable 5. Descriptive data with BMI.

| BMI | Frequency | Percent | Cumulative Frequency | Cumulative Percent |
| --- | --- | --- | --- | --- |
| <25 | 59123 | 52.96 | 59123 | 52.96 |
| 25-30 | 33314 | 29.84 | 92437 | 82.81 |
| >30 | 19194 | 17.19 | 111631 | 100.00 |
| Frequency Missing = 45615 | | | | |

## eTable 6. Description of mortality and BMI; p<0.0001.

| Table of fallece2meses by imcdef2c | | | |
| --- | --- | --- | --- |
| Death | BMI | | |
| Frequency Percent Row Pct Col Pct | <=30 | >30 | Total |
| No | 90257 80.85 83.09 97.64 | 18368 16.45 16.91 95.70 | 108625 97.31 |
| Yes | 2180 1.95 72.52 2.36 | 826 0.74 27.48 4.30 | 3006 2.69 |
| Total | 92437 82.81 | 19194 17.19 | 111631 100.00 |

## eTable 7. Multilevel analysis including BMI.

| **Variables** | **Univariable analysis** |  | **Multivariable analysis with multilevel analysis** |  |
| --- | --- | --- | --- | --- |
|  | **OR [95%CI]** | **p-value** | **OR [95%CI]** | **p-value** |
| Sex |  |  |  |  |
| Male vs. Female | 1.19 [1.11, 1.27] | 0.0021 | 2.04 [1.89, 2.23] | <0.0001 |
| Age (years) | 1.12 [1.11, 1.12] | <0.0001 | 1.12 [1.11, 1.12] | <0.0001 |
| Physical comorbidities |  |  |  |  |
| Cardiovascular disease | 4.47 [3.93, 5.08] | <0.0001 |  |  |
| Pulmonary disease | 1.44 [1.26, 1.65] | <0.0001 |  |  |
| Metabolic and endocrine disease | 4.84 [4.23, 5.54] | <0.0001 | 1.35 [1.16, 1.58] | 0.0001 |
| Renal disease | 13.26 [11.92, 14.74] | <0.0001 | 1.95 [1.72, 2.21] | <0.0001 |
| Hepatic disease | 3.70 [2.64, 5.18] | <0.0001 |  |  |
| Neurology disorder | 8.05 [6.79, 9.55] | <0.0001 |  |  |
| Cancer | 6.55 [5.88, 7.30] | <0.0001 | 2.28 [2.01, 2.59] | <0.0001 |
| BMI > 30 | 1.862 [1.72, 2.02] | <0.0001 | 1.33 [1.22, 1.46] | <0.0001 |
| Psychiatric disorders |  |  |  |  |
| Substance abuse | 0.91 [0.57, 1.46] | 0.4702 |  |  |
| Anxiety disorder | 0.50 [0.37, 0.68] | <0.0001 |  |  |
| Psychosis | 6.51 [5.05, 8.40] | <0.0001 | 1.43 [1.05, 1.96] | 0.0241 |
| Affective disorder | 0.85 [0.69, 1.06] | 0.1566 |  |  |
| Personality disorders | 1.55 [0.21, 11.49] | 0.9435 |  |  |
| Eating disorders | <0.001 [<0.001, >999.9] | 0.9082 |  |  |
| Others | 2.47 [1.77, 3.45] | <0.0001 |  |  |
